# RORgamma agonists enhance survival and memory of type 17 T cells and improve anti-tumor activity

**DOI:** 10.1186/2051-1426-3-S2-P23

**Published:** 2015-11-04

**Authors:** Xiao Hu, Jacques Moisan, Kinga Majchrzak, Chuck Lesch, Yahong Wang, Brian Sanchez, Xikui Liu, Rodney Morgan, David Mertz, Dick Bousley, Chad van Huis, Don Skalitzky, Clarke Taylor, Thomas Aicher, Peter Toogood, Weiping Zou, Gary Glick, Chrystal Paulos, Laura Carter

**Affiliations:** 1 Lycera Corp, Ann Arbor, MI, USA; 2 Medical University of South Carolina, Charleston, SC, USA; 3 University of Michigan, Ann Arbor, MI, USA

## Background

Enhancing tumor-directed immune responses has emerged as an important therapeutic approach to many cancers. Th17/Tc17 cells can mediate robust anti-tumor responses in rodent models and are associated with improved prognosis in some human cancers. RORgt is the key transcription factor controlling the development and function of these cells by supporting the expression of pro-inflammatory cytokines and survival genes while reducing expression of co-inhibitory receptors such as PD-1.

## Methods

To enhance the function of anti-tumor Type 17 T cells, small molecule RORg agonists were designed. These synthetic agonists, when present during murine and human T cell activation *in vitro*, increase production of IL-17A and other cytokines/chemokines, and improve cell survival after resting or re-activation.

## Results

This improved survival translates to a potent and durable anti-tumor response as adoptive cell therapy using RORg agonist-treated T cells significantly reduces growth of established B16F10 and EG.7 tumors compared to untreated cells. Up to 71 days post transfer, more donor T cells can be recovered from spleens and tumors of mice receiving RORg agonist-treated cells. These cells maintain elevated IL-17 production and reduced co-inhibitory receptor expression suggesting that RORg agonist-induced changes are long-lasting. Interestingly, tumor-specific T cells recovered from mice receiving agonist-treated cells expressed central memory (CD44+CD62L+) or stem-like (CD44-CD62L+) markers vs. the predominantly effector (CD44+CD62L-) cells recovered from animals receiving untreated T cells. In separate experiments, oral administration of RORg agonists significantly inhibits the growth of subcutaneous MC38 and 4T1 tumors in an immune-dependent manner with significantly increased RORg and IL-17 expression in tumors consistent with an increased survival or recruitment of type 17 cells.

## Conclusions

By enhancing cytokine production, decreasing co-inhibitory receptor expression while promoting long term survival and self-renewal of T cells, RORg agonists represent a promising immunotherapy approach for the treatment of cancer.

